# Inducible Nitric Oxide Regulates Brush Border Membrane Na-Glucose Co-transport, but Not Na:H Exchange via p38 MAP Kinase in Intestinal Epithelial Cells

**DOI:** 10.3390/cells7080111

**Published:** 2018-08-19

**Authors:** Palanikumar Manoharan, Shanmuga Sundaram, Soudamani Singh, Uma Sundaram

**Affiliations:** 1 Department of Molecular Genetics, Biochemistry & Microbiology, University of Cincinnati, Cincinnati, OH 45221, USA; girimicro@yahoo.com; 2 Department of Clinical and Translational Sciences and Appalachian Clinical and Translational Science Institute, Joan C. Edwards School of Medicine, Marshall University, Huntington, WV 25701, USA; sundaram1@live.marshall.edu (S.S.); singhs@marshall.edu (S.S.)

**Keywords:** peroxynitrite, SGLT1, p38 MAPK, chronic intestinal inflammation

## Abstract

During chronic intestinal inflammation in rabbit intestinal villus cells brush border membrane (BBM) Na-glucose co-transport (SGLT1), but not Na/H exchange (NHE3) is inhibited. The mechanism of inhibition is secondary to a decrease in the number of BBM co-transporters. In the chronic enteritis mucosa, inducible nitric oxide (iNO) and superoxide production are known to be increased and together they produce abundant peroxynitrite (OONO), a potent oxidant. However, whether OONO mediates the SGLT1 and NHE3 changes in intestinal epithelial cells during chronic intestinal inflammation is unknown. Thus, we determined the effect of OONO on SGLT1 and NHE3 in small intestinal epithelial cell (IEC-18) monolayers grown on trans well plates. In cells treated with 100 μM SIN-1 (OONO donor) for 24 h, SGLT1 was inhibited while NHE3 activity was unaltered. SIN-1 treated cells produced 40 times more OONO fluorescence compared to control cells. Uric acid (1mM) a natural scavenger of OONO prevented the OONO mediated SGLT1 inhibition. Na^+^/K^+^-ATPase which maintains the favorable trans-cellular Na gradient for Na-dependent absorptive processes was decreased by OONO. Kinetics studies demonstrated that the mechanism of inhibition of SGLT1 by OONO was secondary to reduction in the number of co-transporters (V_max_) without an alteration in the affinity. Western blot analysis showed a significant decrease in SGLT1 protein expression. Further, p38 mitogen-activated protein (MAP) kinase pathway appeared to mediate the OONO inhibition of SGLT1. Finally, at the level of the co-transporter, 3-Nitrotyrosine formation appears to be the mechanism of inhibition of SGLT1. In conclusion, peroxynitrite inhibited BBM SGLT1, but not NHE3 in intestinal epithelial cells. These changes and the mechanism of SGLT1 inhibition by OONO in IEC-18 cells is identical to that seen in villus cells during chronic enteritis. Thus, these data indicate that peroxynitrite, known to be elevated in the mucosa, may mediate the inhibition of villus cell BBM SGLT1 in vivo in the chronically inflamed intestine.

## 1. Introduction

In the mammalian small intestine, glucose is primarily absorbed by Na-glucose co-transport (SGLT1; *Slc5a1*). The glucose transporter SGLT1 uses a Na^+^ gradient to transport Na^+^ and glucose at a 2:1 stoichiometric ratio against a glucose gradient [1]. In each cycle, a sugar molecule is co-transported with Na^+^ across the cell, which is accompanied by 260 water molecules [2]. This mechanism was calculated to account for five L of water absorbed per day in the human intestine and formed the molecular basis of oral rehydration therapy aimed to control mortality associated with cholera and other infectious diarrheal diseases [3].

Coupled NaCl absorption occurs via the dual operation of Na:H (NHE3; *Slc9a3*) and Cl:HCO_3_ (Down Regulated in Adenoma (DRA; *Slc26a3*) or Putative Anion Transporter 1 (PAT1; *Slc26a6*)) exchange. All of these essential transport processes are found on the brush border membrane (BBM) of absorptive villus, but not secretory crypt cells in the mammalian small intestine. The favorable transcellular Na-gradient for SGLT1 and NHE3 is provided by Na/K-ATPase on the basolateral membrane (BLM). 

In human inflammatory bowel disease (IBD), malabsorption of electrolytes, nutrients, and water leads to the most common and disabling morbidities of the disease, namely diarrhea and malnutrition [4,5,6,7]. In a rabbit model of IBD, resembling the human condition, the inhibition of coupled NaCl and Na-glucose co-transport in the small intestine has been previously described [8,9]. The inhibition of SGLT1 at the level of intact villus cells was secondary to both a reduction in BBM co-transporter activity as well as decreased Na^+^/K^+^-ATPase activity in the BLM. At the level of the co-transporter in the BBM, the mechanism of inhibition of SGLT1 was secondary to a reduction in the synthesis of SGLT1 protein without an alteration in the affinity of the co-transporter for glucose.

The inhibition of coupled NaCl absorption during IBD was secondary to a reduction in Cl:HCO_3_ exchange activity without a change in Na:H exchange. Further, inhibition of Cl:HCO_3_, namely, DRA, was secondary to a reduction in the affinity of the exchanger for Cl without an alteration in the number of DRA exchangers in the villus cell BBM during chronic intestinal inflammation [10]. 

In the chronically inflamed intestine inducible nitric oxide (iNO) levels are dramatically increased secondary to increased iNO synthase (iNOS) activity. Likewise, reactive metabolites of oxygen such as superoxide are also known to be increased [11,12,13]. In these conditions, nitric oxide reacts with superoxide to form peroxinitrate (OONO) a potent nitrating and oxidizing radical that has been proposed to contribute to a wide variety of pathophysiological alterations in inflammatory diseases including IBD. The biological targets of OONO include lipids, thiols, DNA, and tyrosine residues [14].

Increased expression of iNOS and elevated levels of OONO have been demonstrated in patients with Crohn’s disease and ulcerative colitis. Moreover, 3-nitrotyrosine (3-NT) formation serves as a foot print or evidence for OONO production and they are found in clinical biopsy samples from IBD patients [15,16,17] and in animal models of chronic intestinal inflammation [18]. Peroxynitrite is difficult to measure in vivo and in vitro due to its short half-life (1.9 sec) at physiological pH, hence 3-nitrotyrosine (3-NT) formation [19] and peroxynitrite-dependent oxidation of dihydrorhodamine 123 to rhodamine [20,21] is used as the standard method for measuring OONO formation. OONO has been reported to activate mitogen-activated protein kinase (MAPK) [22] which has been shown to regulate intestinal transport processes and inhibiting MAPK signaling pathways revealed beneficial effects on ulcer healing in patients with Crohn’s disease [23,24].

As previously noted in a rabbit model of IBD, unique changes in coupled NaCl absorption and Na-glucose co-transport have been demonstrated. However, the effect of OONO on NHE3 and SGLT1 in intestinal epithelial cells is unknown. Therefore, the current study was designed to investigate the effect and regulatory mechanism of OONO donor on SGLT1 and NHE3 activity in intestinal epithelial cells (IEC-18) and determine the signaling mechanism.

## 2. Materials and Methods

### 2.1. Tissue Culture and Drug Treatment

Rat IEC-18 were grown in high glucose Dulbecco's modified Eagle’s medium (Gibco, Gaithersburg, MD, USA) supplemented with 2 U/mL of insulin, 0.5 mM β-hydroxybutyrate, and 10% fetal calf serum (Atlanta Biologicals, Flowery Branch, GA, USA) and incubated at 37 °C with 10% CO_2_. Cells were grown on trans well plates and were used at 10 days post confluence. IEC-18 cells were treated with SIN-1 chloride (OONO donor; 100 μM) or vehicle and the experiments were performed 24 h post treatment. Uric acid (Sigma) and selective inhibitors for p38 MAPK were used to treat the IEC-18 cells 1 h prior to SIN-1 treatment. Cell signaling inhibitors were obtained from EMD chemicals and the SIN-1 from Calbiochem (Burlington, MA, USA) and, all the other chemicals were purchased from Sigma Aldrich (St. Louis, MO, USA).

### 2.2. Na-Glucose Cotransport in IEC-18 Cells

The 10 days post confluent IEC-18 cells were washed and incubated with Leibowitz-15 medium supplemented with 10% fetal bovine serum and incubated for 1 h. The cells were then washed and incubated with Na-free medium containing 130 mM trimethyl ammonium chloride (TMACl), 4.7 mM KCl, 1.2 mM KH_2_PO_4_, 1 mM MgSO_4_, 1.25 mM CaCl_2_, 20 mM HEPES and gassed with 100% O_2_ (pH 7.4 at 37 °C) for 10 min. Uptakes were performed at desired time intervals in reaction medium containing either 130 mM NaCl or 130 mM TMACl in HEPES medium (as described above) with 10 µCi of ^3^H-*O*-methyl glucose (OMG) and 100 µM cold OMG. Cells were then washed with cold Na-free medium and then incubated with 1N NaOH for 20 min at 70 °C before addition of 4 mL of scintillation fluid (Ecoscint; National Diagnostics, Atlanta, GA, USA). Radioactivity was determined in a Beckman 6500 Beta scintillation counter (Beckman, Indianapolis, IN, USA).

### 2.3. Na^+^/H^+^ Exchange in IEC-18 Cells

IEC-18 cells at 10 days post confluence were washed with Leibowitz-15 medium. The cells were then incubated with Na free medium for 10 min. Uptakes were performed at desired time intervals in reaction medium containing 130 mM TMACl HEPES medium, 10 µCi of ^22^Na, 1 mM NaCl with and without 50 µM EIPA to determine Na^+^/H^+^ exchange in IEC-18 cells. These cells were then washed with ice cold Na-free buffer and processed as described above.

### 2.4. Na^+^/K^+^-ATPase Assay

Na^+^/K^+^-ATPase activity was measured by quantifying the ouabain-sensitive rate of ATP-hydrolysis in cultured IEC-18 cells. The assay was performed in control and SIN-1 treated IEC-18 cell homogenates as previously described [25,26]. Enzyme specific activity was expressed as nanomoles of P_i_ released per milligram of protein per minute. 

### 2.5. Peroxynitrite Measurement

Peroxynitrite was measured as fluorescence produced from oxidation of dihydrorhodamine 123 (DHR). Briefly, the IEC-18 cells were grown into confluent monolayers in 24 well plates. These cells were treated with 100 μM of SIN-1 along with 10 μM of DHR and incubated for 90 min. DHR fluorescence was measured with Perkin-Elmer 650-40 spectroflurometer (Perkin Elmer, San Jose, CA, USA) at excitation and emission wavelength of 485 and 520 nm, respectively.

### 2.6. Western Blot

Brush border membrane (BBM) from IEC-18 cells for Western blot analysis was prepared as described earlier [27]. BBM and total cell lysate of IEC-18 cells were solubilized in RIPA buffer (Santa Cruz, Dallas, TX, USA) containing Halt protease inhibitor cocktail (Pierce, Thermo Scientific, Waltham, MA, USA) and cellular protein concentration was measured at A280 nm. Equal amounts of protein samples (150 µg) were resolved using 10% SDS PAGE and immobilized to Immobilon-P membranes (Millipore, Burlington, MA, USA). These membranes were probed with SGLT1 (Abcam, Cambridge, MA, USA), Ezrin (Millipore, Burlington, MA, USA), p38 MAPK, and phos p38 MAPK (Cell Signaling) specific antibodies at 1:1000 dilutions. Secondary detection using goat anti-rabbit Alexa 680 (Invitrogen, Carlsbad, CA, USA) was performed at 1:10,000 dilutions. Membranes were scanned using Odyssey Infrared Imaging System (LICOR Bioscience, Lincoln, NE, USA).

### 2.7. Immunocytochemistry

Immunocytochemistry for 3-NT formation was performed as described by Coon et al., [28]. Briefly, IEC-18 cells were transferred to a coverslip and fixed in 4% (*v*/*v*) paraformaldehyde for 20 min. Antigen retrieval was performed by incubating the cells with 0.5% Triton X-100 (Sigma-Aldrich, St. Louis, MO, USA) in phosphate buffered saline (PBS) for 2 min at room temperature. The cells were then incubated with 1:500 diluted anti-rabbit SGLT1 (Abcam, Cambridge, MA, USA) and 1:250 diluted anti-mouse nitrotyrosine primary antibodies overnight at 4 °C. The next day cells were washed with PBS and incubated with secondary antibodies, Alexa fluor 488 goat anti-rabbit and Alexa fluor 555 donkey anti-mouse (Invitrogen, Carlsbad, CA, USA) to detect SGLT1 and nitro tyrosine formation respectively. ProLong Gold Antifade Reagent (Invitrogen, Carlsbad, CA, USA) was used to mount the cells and the fluorescence signals generated were observed under a Zeiss LSM 510 confocal microscope.

### 2.8. Immunoprecipitation

The control and SIN-1 treated IEC-18 cell lysates were subjected to immunoprecipitation with 10 μg of anti-nitro tyrosine antibody (Abcam, Cambridge, MA, USA). Pierce classic IP kit was used to immobilize the anti-nitrotyrosine IgG to the resin and the immunoprecipitated proteins were eluted with Laemmli sample buffer. The samples obtained from immunoprecipitation were subjected to immunoblotting and probed for SGLT1. The secondary detection was performed as described in Western blot analysis and images were recorded.

### 2.9. Data Presentation

Results presented represent means ± SEM of the experiments performed and calculated by the GraphPad Instat (La Jolla, CA, USA) program. Student’s t-test was performed for statistical analysis. 

## 3. Results

### 3.1. Effect of OONO on SGLT1 Activity in IEC-18 Cells

**Figure 1:**
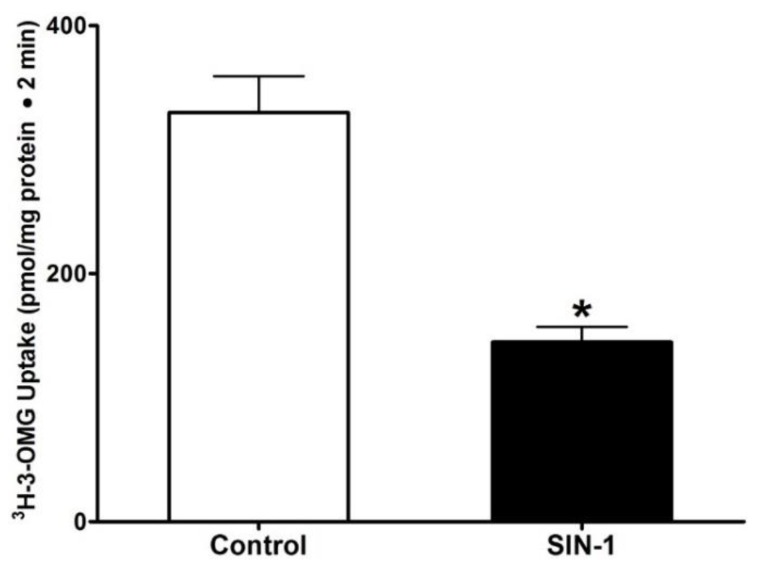
Effect of peroxynitrite on SGLT1 in intestinal epithelial cells (IEC-18). Na-dependent 3H-O-Methyl glucose uptake (SGLT1) was present in IEC-18 cells. SGLT1 activity was markedly diminished when the cells were treated with SIN-1. n = 3, * *p* < 0.01.

The uptake of 3-OMG was done in the presence and absence of extracellular Na in IEC-18 cells and Na stimulated 3-OMG uptake was measured as SGLT1 activity. Then SIN-1 treatment was used to demonstrate the effect of OONO on activity Na-glucose co-transport. As shown in Figure 1, SGLT1 was significantly inhibited by OONO in intact IEC-18 cells (329 ± 29 pmol/mg•protein/2 min in control and 144.8 ± 12* pmol/mg•protein/2 min in SIN-1 treated cells, n = 3, * *p* < 0.01). These data demonstrated that SGLT1, determined as Na-dependent 3-OMG uptake was significantly inhibited by OONO produced by SIN-1 treatment.

### 3.2. Effect of OONO on Na^+^/H^+^ Exchange IEC-18 Cells

**Figure 2:**
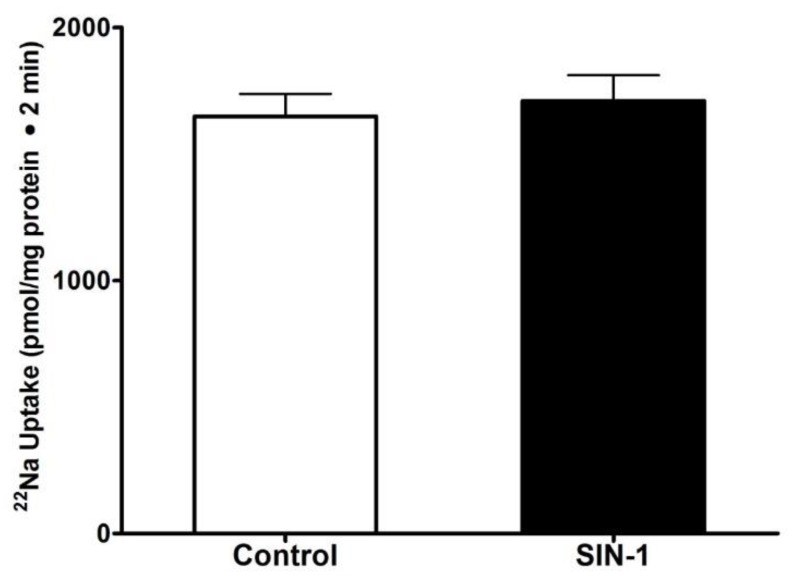
Effect of peroxynitrite on Na/H exchange (NHE3) in IEC-18 cells. A proton gradient-driven EIPA sensitive Na uptake was present in IEC-18 cells. Na-H exchange remained unaltered in the SIN-1 treated IEC-18 cells. n = 3.

We next looked at the effect of peroxynitrite on Na/H exchange in IEC-18 cells. H^+^ dependent, EIPA sensitive ^22^Na uptake was measured as NHE3 activity. In contrast to SGLT1, SIN-1 treatment did not alter the NHE3 activity in IEC-18 cells (Figure 2). These data indicate that Na/H exchange is present in IEC-18 cells but was unaltered by OONO.

### 3.3. Effect of OONO on Na^+^/K^+^-ATPase Activity in IEC-18 Cells

**Figure 3:**
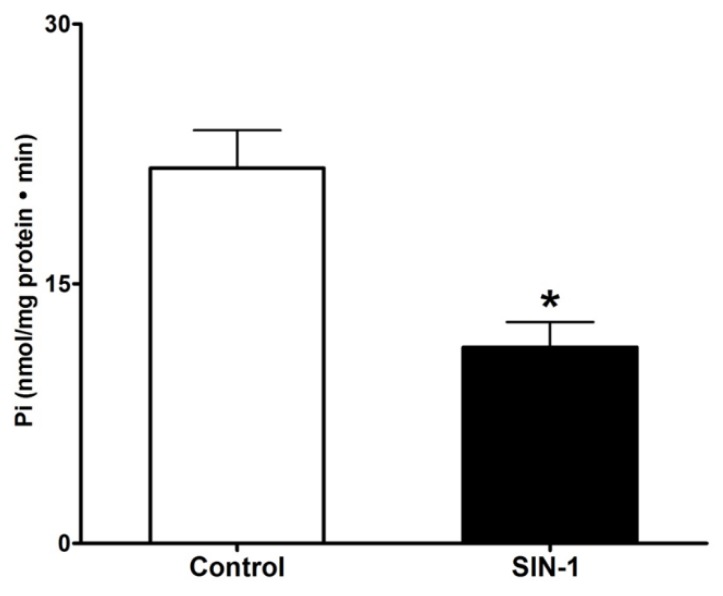
Effect of peroxynitrite on Na^+^/K^+^-ATPase activity in IEC-18 cells. Na+/K+-ATPase activity measured as an inorganic phosphate released was significantly diminished by SIN-1 treatment in IEC-18 cells. n = 3, * *p* < 0.02.

Intracellular Na homeostasis is important for the functioning of BBM Na transport processes. Then, we next looked at the effect of SIN-1 treatment on Na^+^/K^+^-ATPase activity in IEC-18 cells. As shown in Figure 3. SIN-1 treatment significantly diminished the Na^+^/K^+^-ATPase activity (21.67 ± 2.16 and 11.33 ± 1.45 nmol/mg protein•min in control and SIN-1 treated cells respectively). Thus, at the level of the intact cell, inhibition of SGLT1 by OONO, may at least, in part, be secondary to altered Na extruding capacity of cells.

### 3.4. Uric Acid Prevents Effect of OONO on SGLT1 Activity

**Figure 4:**
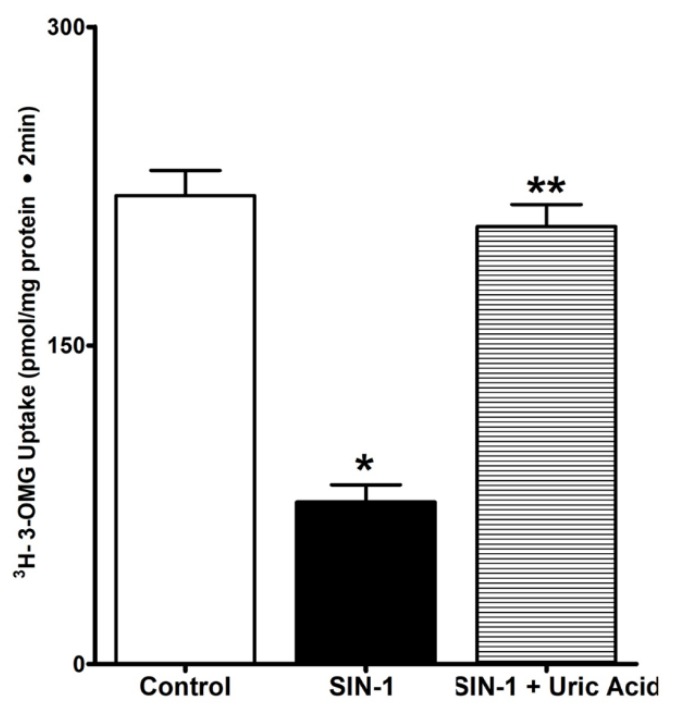
Uric acid prevents abundant peroxynitrate (OONO) effect on SGLT1 activity. SIN-1 treatment significantly diminished the SGLT1 activity in IEC-18 cells. Uric acid, a natural scavenger for peroxynitrite, prevented the effect of SIN-1 treatment on SGLT1 activity in IEC-18 cells. Thus, these data show that the inhibition of SGLT1 activity was due to peroxynitrite produced from SIN-1 treatment. n = 3, * *p* < 0.01 (control Vs SIN-1), ** *p* < 0.05 (SIN-1 vs. SIN-1 + Uric acid).

Uric acid is a well-known natural scavenger of OONO. We next looked at whether uric acid could prevent the OONO mediated SGLT1 inhibition in IEC-18 cells. As shown in Figure 4, uric acid reversed the inhibition of SGLT1 by OONO in IEC-18 cells. These data indicate that uric acid can successfully scavenge OONO and hence the SGLT1 activity remains unaltered in SIN-1 treated cells.

### 3.5. Measurement of OONO

**Figure 5:**
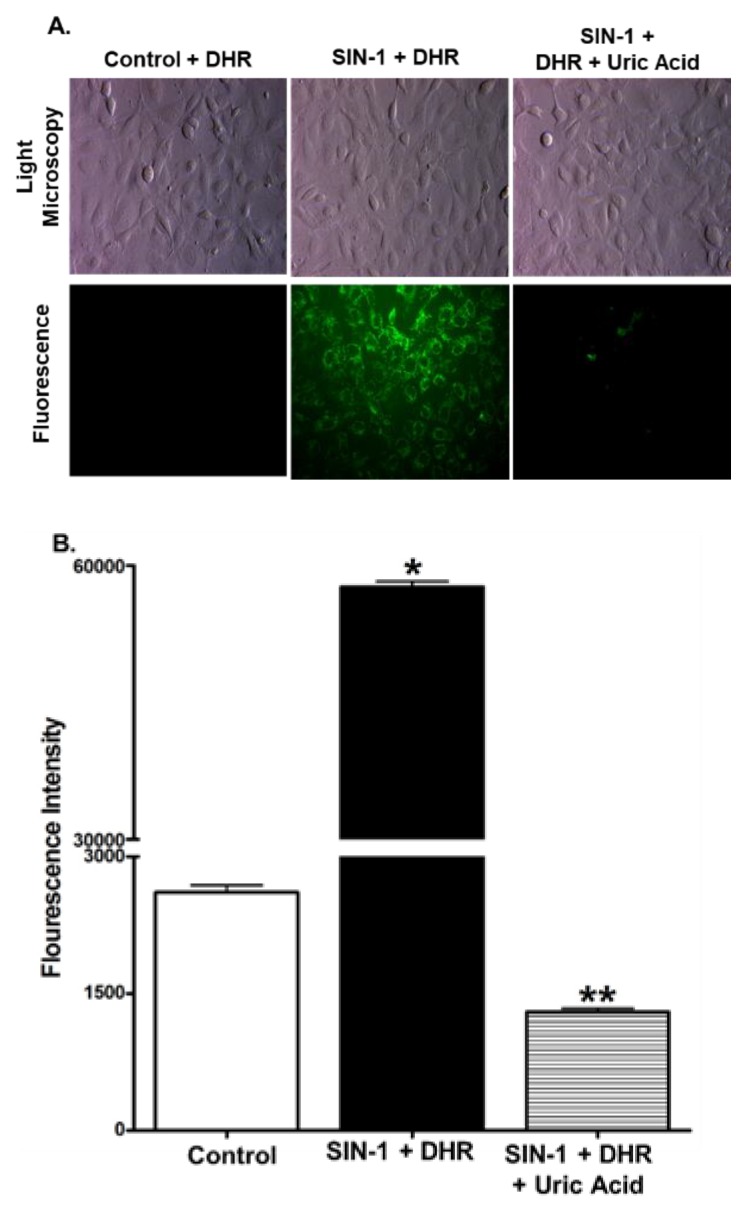
Dihydrorhodamine 123 oxidation by SIN-1 treatment in IEC-18 cells. (**A**) Dihydrorhodamine was oxidized by peroxynitrite produced from SIN-1 and therefore flourescence was observed in IEC-18 cells. (**B**) Intensity of the fluorescence produced due to oxidization of Dihydrorhodamine was significantly elevated in SIN-1 treated cells since uric acid prevents the oxidation there was no fluorescence measured in that condition. n = 4; * *p* < 0.001 (control Vs SIN-1), ** *p* < 0.01 (SIN-1 vs. SIN-1 + Uric acid).

We next demonstrated the production of OONO in SIN-1 treated IEC-18 cells. OONO is known to oxidize DHR into a fluorescent end product and the fluorescence emitted was measured in IEC-18 cells. DHR was oxidized in SIN-1 treated IEC-18 cells reflecting an increase in fluorescence of approximately 40-fold and the oxidization was significantly prevented by uric acid (Figure 5).

### 3.6. Kinetic Studies for SGLT1 Activity

**Figure 6:**
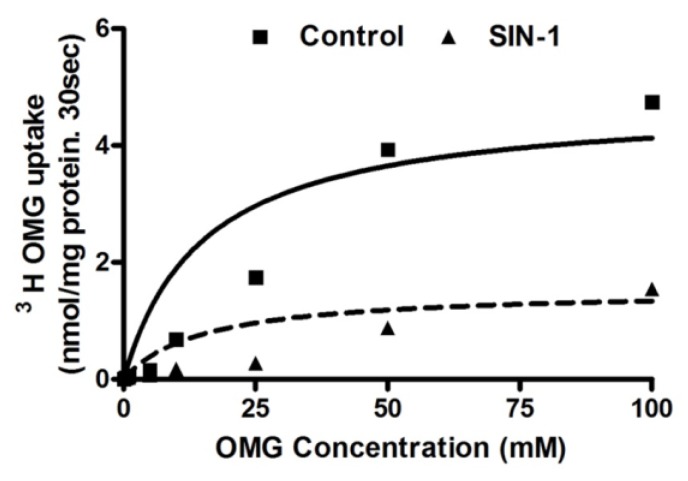
Kinetic parameters for SGLT1 activity in SIN-1 treated IEC-18 cells. Na-dependent glucose uptake is shown as a function of varying concentrations of extravesicular glucose. Isosmolarity was maintained by adjusting the concentration of mannitol. Uptake for all concentrations was determined at 30 s. As the concentration of extravesicular glucose was increased, uptake of glucose was stimulated and subsequently became saturated in IEC-18 cells in all conditions. The maximal rate of uptake of glucose (V*_max_*) was significantly reduced by SIN-1 treatment but the affinity (1/K*_m_*) for glucose uptake was unaffected in the SIN-1 treated IEC-18 cells. (n = 3, * *p* < 0.05).

To determine the mechanism of OONO mediated inhibition of SGLT1, kinetic studies were performed. Na-dependent glucose uptake is shown as a function of increasing concentrations of extra cellular glucose in IEC-18 cells. As the concentration of extra-cellular glucose was increased, the uptake of Na-dependent glucose was stimulated and subsequently became saturated in all conditions Figure 6. The affinity (*K_m_*) for glucose uptake was not altered by SIN-1 treatment (5.2 ± 0.5 mM in control and 4.8 ± 0.3 mM in SIN-1 treated IEC-18 cells). However, the maximal rate of uptake (*V_max_*) of glucose was significantly reduced in SIN-1 treated cells compared to control (*V_max_* was 4.5 ± 0.8 and 1.5 ± 0.2 nmol/mg protein 30 s in control and SIN-1 treatment respectively, n = 3; * *p* < 0.05). Thus, these data indicated that the mechanism of inhibition of SGLT1 by OONO was secondary to reduction in the number of co-transporters without an alteration in the affinity of the co-transporter for glucose. 

### 3.7. SGLT1 Protein Expression

**Figure 7:**
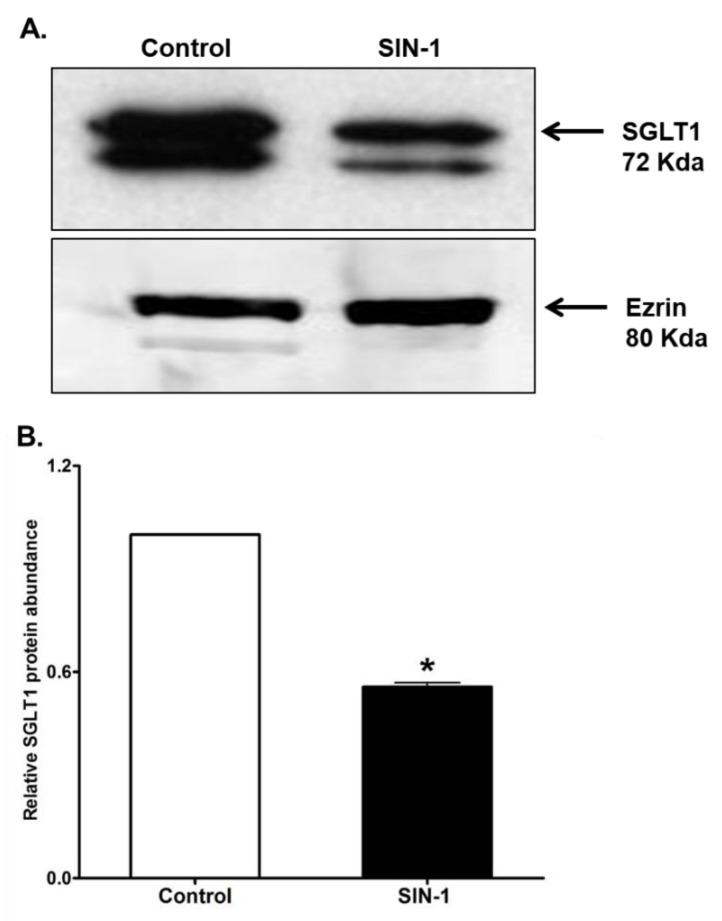
Effect of peroxynitrite on SGLT1 protein expression. (**A**) Representative Western blot of SGLT1 protein expression that is significantly diminished by SIN-1 in IEC-18 cells. (**B**) Densitometry analysis showed that SGLT1 proteins levels are significantly diminished when treated with SIN-1 compared to the control. (n = 3; * *p* < 0.001). Control is assigned a value of 1, and the SIN-1 value is relative to the control. Error bars when not visible are inclusive in the figure.

SGLT1 mediates the Na^+^-glucose co-transport in intestinal epithelial cell BBM. Western blot performed in BBM of IEC-18 cells using rat specific SGLT1 antibody demonstrated that SIN-1 treatment diminished the immunoreactive protein levels of SGLT1 Figure 7A. Densitometry quantitation substantiated these findings Figure 7B. These data in conjunction with the kinetic studies above indicated that the mechanism of inhibition of SGLT1 by OONO was secondary to reduction in BBM co-transporter numbers rather than an alteration in the affinity of the co-transporter for glucose. 

### 3.8. p38 MAPK Signaling

**Figure 8:**
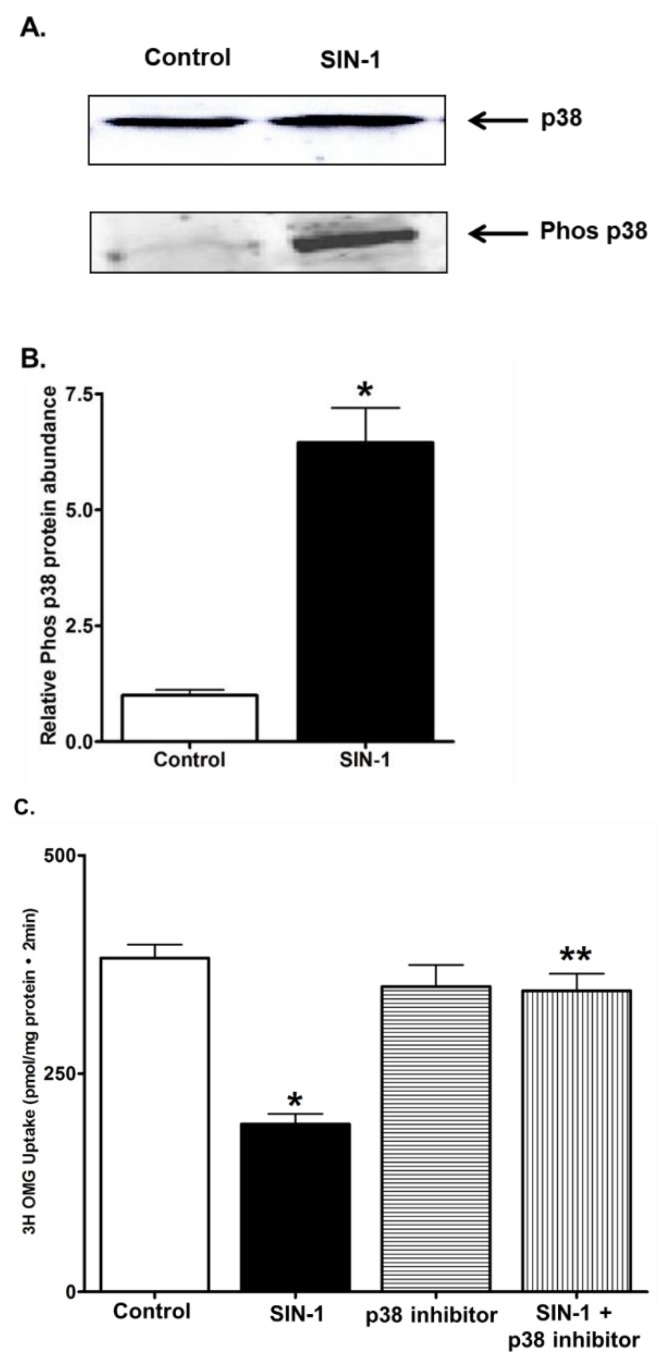
Peroxynitrite activated p38 mitogen-activated protein (MAP) kinase inhibits function of SGLT1. (**A**) Representative Western blot for endogenous p38 and OONO activated phosphorylated p38 expression levels in SIN-1 treated IEC-18 cells. (**B**) Densitometry analysis for phosphorylated p38 levels between control and SIN-1 treatment (n = 4, * *p* < 0.001). (**C**) Peroxynitrite treatment in the presence of p38 MAP kinase inhibitor did not affect the SGLT1 mediated Na-glucose co-transport in IEC-18 cells. n = 4, * *p* < 0.01 (control vs. SIN-1 treatment), ** *p* < 0.05 (SIN-1 vs. SIN-1 + p38 MAPK inhibitor).

We next determined the role of p38 MAP kinase signaling pathway in the OONO mediated SGLT1 inhibition. Incubation of IEC-18 cells with 100µM SIN-1 induced transient phosphorylation of p38 MAPK (Figure 8A,B). Then, we performed 3-OMG uptake in IEC-18 cells treated with SIN-1 in the presence of p38 specific inhibitor. The 3-OMG uptake was not altered by SIN-1 in the presence of p38 inhibitor (Figure 8C). Other inhibitors for PKCα, AKT, CamKII, and PI3K did not protect the OONO effect on SGLT1 activity (Data not shown). These data showed that SGLT1 activity is selectively inhibited by phosphorylation p38 MAPK pathway during SIN-1 treatment.

### 3.9. 3-Nitrotyrosine Formation

**Figure 9:**
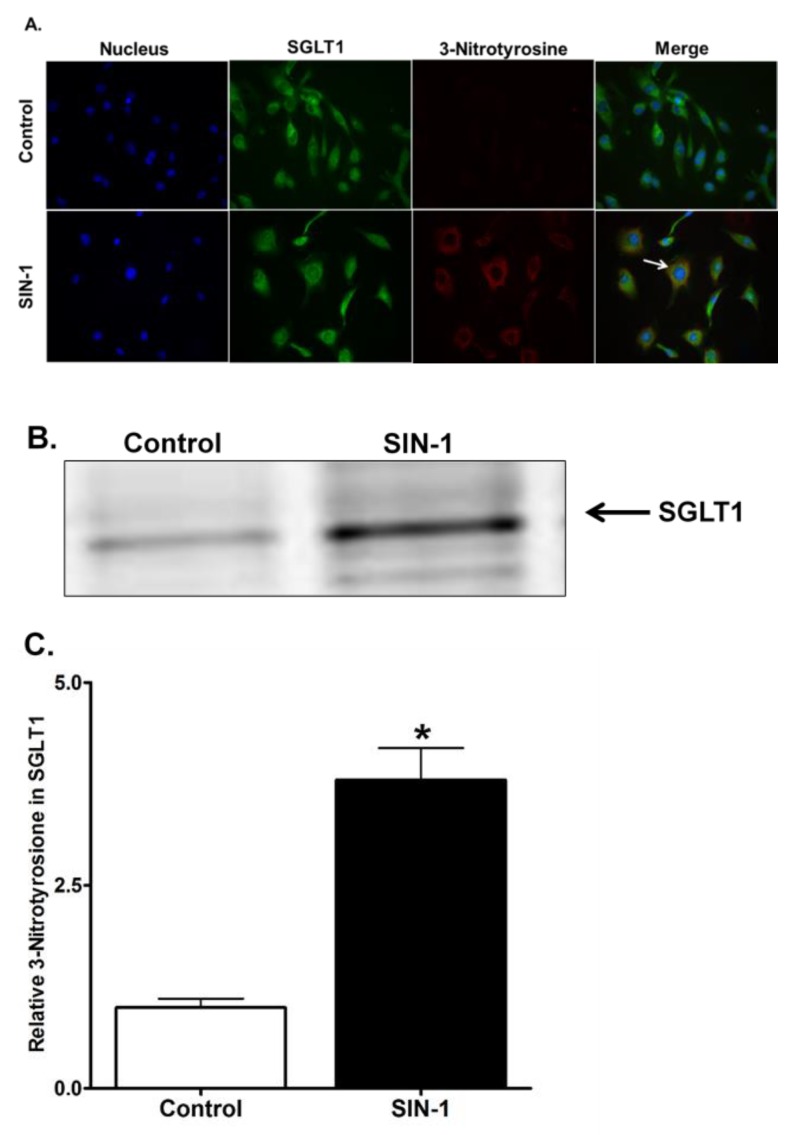
Peroxynitrite induced 3-NT formation in SGLT1. (**A**) Immunohistochemistry analysis reveals that SIN-1 treatment increased tyrosine nitration in IEC-18 cells compared to control. Merged picture of SGLT1 and anti-tyrosine staining showed co-localization of both the proteins. (**B**) Representative Western blot analysis of immunoprecipitated samples showing an increase in OONO induced tyrosine nitration of SGLT1 when treated with SIN-1 compared to control. (**C**) Densitometry analysis showed that the 3-NT formation was significantly increased in SGLT1 after SIN-1 treatment in IEC-18 cells (n = 4; * *p* < 0.05). Control is assigned a value of 1, and the SIN-1 value is relative to the control.

To determine the formation of 3-NT in the IEC-18 cells, we next performed immunocytochemistry in SIN-1 treated cells. 3-NT formation was observed in SIN-1 treated IEC-18 cells (Figure 9A). In addition, co-localization of SGLT1 and 3-NT was evident from immunofluorescence staining. To further confirm the formation of 3-nitrotyrosine in SGLT1, we performed immunoprecipitation analysis. The IEC-18 cell lysates that were immunoprecipitated with anti-nitrotyrosine antibody and immuno probed for SGLT1, clearly demonstrated SGLT1 is post-translationally modified by tyrosine nitration in SIN-1 treated samples (Figure 9B). Densitometry quantitation substantiated our finding that 3-NT formation in SGLT1 in SIN-1 treated samples is significantly increased (Figure 9B). These results provide evidence that OONO modifies tyrosine residues of SGLT1, thus inhibiting the transport activity in IEC-18 cells.

## 4. Discussion

In a rabbit model of chronic intestinal inflammation resembling human inflammatory bowel disease, multiple unique alterations in electrolyte and nutrient transport processes have been demonstrated [8,29,30,31,32]. Interestingly, of the primary Na absorptive pathways, while Na-glucose co-transport was diminished in the chronically inflamed intestine, Na:H exchange was unaffected. At the cellular level SGLT1 was inhibited both secondary to reduced SGLT1 activity on the BBM as well as secondary to diminished activity of BLM Na^+^/K^+^-ATPase, which provides the favorable Na-gradient for SGLT1. Further, the molecular mechanism of inhibition of SGLT1 appears to be diminished *de novo* synthesis of SGLT1 as compared to altered trafficking to the BBM. In contrast, Na:H exchange, which along with Cl:HCO_3_ promotes coupled NaCl absorption, was not affected in the chronically inflamed intestine. Immune-inflammatory mediators and reactive metabolites of oxygen and nitrogen are known to be present in abundance in the mucosa during chronic inflammation and are thought to potentially mediate the alterations in intestinal transporters.

Nitric oxide is a very biologically active molecule known to affect intestinal transport processes in health and disease. In the normal intestine constitutive nitric oxide (cNO) has been shown to affect absorption and secretion. Whereas in the inflamed intestine, orders of magnitude greater levels of NO produced by inducible nitric oxide synthase (iNOS), termed inducible nitric oxide (iNO), is thought to have deleterious effects on intestinal transport. For example, in the former case, previous studies have demonstrated that the intestinal epithelial cell brush border membrane primary Na absorptive pathways, namely SGLT1 and NHE3, are uniquely regulated by constitutive nitric oxide (cNO). When cNO was reduced, NHE3 was inhibited while SGLT1 was stimulated. The mechanism of alteration of these transport processes were equally unique: whereas NHE3 inhibition was secondary to a reduction in BBM transporter numbers, SGLT1 stimulation was secondary to an increase in the affinity of the co-transporter for glucose.

While the importance of iNO in inflammation induced tissue injury has been well described, the effect of iNO on SGLT1 and NHE3 is less well understood. Enhanced iNOs expression and subsequent NO production has been reported in patients with IBD [17]. In fact, the deleterious effect is caused due to OONO produced from reaction of NO with superoxide. Indeed, OONO generation has been shown to be markedly increased both in animal models intestinal inflammation as well as in human samples of IBD [33,34].

In the present study the activity of SGLT1 was significantly inhibited by OONO in IEC-18 cells, while the NHE3 activity remained unchanged. At the cellular level OONO mediated inhibition of SGLT1 was secondary to an effect at the co-transporter in the BBM as well as secondary to diminished Na-extruding capacity of the cell. At the co-transporter level in the BBM, kinetics and Western blot studies showed that the OONO mediated inhibition of SGLT1 was secondary to reduction in the number of co-transporters without an alteration in the affinity of the co-transporter for glucose. These changes in SGLT1 and Na/KATPase and unaffected NHE3 were similar to the observations in villus cells from the chronically inflamed intestine in the rabbit model of IBD. Further, the specific mechanisms of alteration of SGLT1, namely the inhibition secondary to a decrease in the number of co-transporters without an alteration in affinity for glucose is also identical. Hence, one explanation is that OONO generated during chronic intestinal inflammation in the rabbit model mediated the alterations in SGLT1.

OONO mediated nitration of proteins is now well documented to affect protein structure and function, resulting in altered cytoskeletal organization and cell signal transduction. For example, OONO mediated nitration of α1 and β1 subunits of renal Na^+^/K^+^ ATPase inhibited their pump activity [35] suggesting the cause for reduction of Na^+^/K^+^ ATPase activity in OONO treated IEC-18 cells is also by nitration. Further, uric acid, a purine metabolite and natural scavenger of OONO, inhibits nitration in vitro and in vivo [36,37]. In this study, uric acid prevented OONO mediated oxidation of DHR123 (Figure 4) and inhibition of SGLT1 activity. These data further indicate that OONO mediated inhibition of SGLT1 is secondary to nitration.

P38 MAPK, a family of MAPKs, is activated by a wide range of environmental stress and proinflammatory cytokines. OONO has been shown to activate p38 MAPK within minutes after treatment. Indeed, we examined the ability of OONO to phosphorylate p38 in IEC18 cells and found rapid increase in the amount of phospho-p38 (Figure 8A). Furthermore, p38 MAPK specific inhibitor blocked the effect of OONO on SGLT1 in IEC-18 cells. Thus, it likely that activated p38 MAPK might serve as a potential signaling molecule which mediates the effect of OONO on SGLT1.

## 5. Conclusions

In conclusion, Na^+^-glucose co-transporter, SGLT1, is inhibited by OONO in IEC18 cells by a mechanism similar to that seen in rabbit chronic intestinal inflammation. Further, these in vitro studies provided additional mechanistic information regarding the regulation of SGLT1 by OONO that may occur during chronic intestinal inflammation. Thus, these data indicate that peroxynitrite, known to be elevated in the mucosa, most likely mediates the inhibition of villus cell BBM SGLT1 in vivo in the chronically inflamed intestines. Furthermore, SGLT1 activity is selectively inhibited by p38 MAP kinase pathway, and our novel finding may be a useful therapeutic target to alleviate the nutrient transporter function during chronic intestinal inflammation.
